# Modeling and Analysis of Dispersive Propagation of Structural Waves for Vibro-Localization

**DOI:** 10.3390/s24237744

**Published:** 2024-12-04

**Authors:** Murat Ambarkutuk, Paul E. Plassmann

**Affiliations:** The Bradley Department of Electrical and Computer Engineering, Virginia Tech, Blacksburg, VA 24061, USA; murata@vt.edu

**Keywords:** occupant localization, sensor fusion, structural vibration, wave propagation, dispersion

## Abstract

The dispersion of structural waves, where wave speed varies with frequency, introduces significant challenges in accurately localizing occupants in a building based on vibrations caused by their movements. This study presents a novel multi-sensor vibro-localization technique that accounts for dispersion effects, enhancing the accuracy and robustness of occupant localization. The proposed method utilizes a model-based approach to parameterize key propagation phenomena, including wave dispersion and attenuation, which are fitted to observed waveforms. The localization is achieved by maximizing the joint likelihood of the occupant’s location based on sensor measurements. The effectiveness of the proposed technique is validated using two experimental datasets: one from a controlled environment involving an aluminum plate and the other from a building-scale experiment conducted at Goodwin Hall, Virginia Tech. Results for the proposed algorithm demonstrates a significant improvement in localization accuracy compared to benchmark algorithms. Specifically, in the aluminum plate experiments, the proposed technique reduced the average localization precision from 7.77 cm to 1.97 cm, representing a ∼74% improvement. Similarly, in the Goodwin Hall experiments, the average localization error decreased from 0.67 m to 0.3 m, with a ∼55% enhancement in accuracy. These findings indicate that the proposed approach outperforms existing methods in accurately determining occupant locations, even in the presence of dispersive wave propagation.

## 1. Introduction

Vibro-localization is an occupant localization method that employs the ambient measurements of structural waves generated by occupants’ activities to determine their locations in a building. Vibro-localization is particularly useful in smart buildings, where the occupants’ locations are used to improve the safety, security, and energy efficiency of the building. For instance, in an emergency situation, the occupants’ locations can be used to guide them to safety, or to help the first responders to locate them. Another example is the monitoring of the gait parameters of the occupants, which can be used to detect the early signs of many neurodegenerative diseases such as Parkinson’s disease, Alzheimer’s disease, and multiple sclerosis [1,2]. Various spatiotemporal characteristics of human gait, such as symmetry [3,4,5,6] and gait variability [7,8,9], which can be obtained with vibro-localization techniques, have been shown to be reliable indicators of neurodegenerative diseases.

Vibro-localization techniques are based on the premise that the structural waves generated by the occupants’ activities propagate through the building’s structure and are measured by a network of sensors, i.e., accelerometers. However, these waves are subject to various propagation phenomena, such as dispersion, attenuation, and reflection, which can transform the waveforms and introduce errors in the localization outcomes. Therefore, each sensor’s measurement corresponds to a transformed version of the wave generated by the occupant, making the localization problem challenging. Dispersion is a particularly challenging phenomenon that affects the wave propagation in real-world settings, i.e., defined by a long propagation path, low-frequency waves, low spectral resolution, etc.

### 1.1. Relevant Literature

The literature in vibro-localization considering dispersion is diverse and spans various disciplines, including structural engineering, signal processing, and machine learning. In their work, Mirshekari et al. [10] proposed a localization technique that employs a two-fold strategy to mitigate the effect of dispersion on the localization outcomes: (1) wavelet-based dispersion mitigation by narrow-band filtering; and (2) adaptive sensor selection to localize the occupant with closer sensors to reduce the effect of dispersion on the localization outcomes. In their later work, the authors extend their technique to accomodate the variability among the occupants [11] and disturbances along the propagation path [12]. The authors show that their technique outperforms the traditional Time-Difference-of-Arrival (TDoA)-based techniques in terms of localization accuracy and robustness, as the perceived wave velocity is automatically deduced from the floor acceleration data collected by a sensor network.

In highly dispersive, one-dimensional wave propagation scenarios, such as those encountered in railroad tracks and bridge structures, multimodal dispersion significantly influences wave behavior. These environments exhibit multiple interacting wave modes across various frequencies, complicating localization tasks. While our study focuses on vibro-localization under weakly dispersive planar wave propagation, similar principles could be adapted to address these challenges. For instance, research on wave propagation and vibration behavior in railway tracks highlights the complex dynamic performance of high-speed railway systems, emphasizing the need for specialized analysis in such contexts [13]. Additionally, studies on ultrasonic guided-wave approaches for detecting wire breaks in bridge cables demonstrate the application of guided-wave modes in multi-wire bridge cables, providing insights into handling multimodal dispersion [14]. However, the algorithms presented in these references are specifically designed for wave propagation analysis and are not directly applicable to the vibro-localization problem discussed in this manuscript.

The Time-of-Arrival (ToA) of flexural waves in a dispersive medium exhibits a nonlinear dependence on the distance *d*, specifically proportional to $d^{4/3}$ [15]. The perceived velocity of the wave, $v_{p}$, at any given distance *d* from the source, is defined based on the ToA of the maximum signal envelope. This relationship is mathematically expressed as $v_{p}\left(d\right)=\frac{1}{\eta }d^{-1/3}$, where η is a constant that depends on the boundary conditions and material properties. This equation indicates that in a dispersive media, the flexural wave’s perceived velocity $v_{p}$ is not constant, but rather decreases nonlinearly as the distance *d* increases. In [15], Bahroun et al. proposed a localization technique that employs the sign of the TDoA measurements to estimate the distance between the source and the sensor [15].

Alajlouni et al. [16] show that dispersive nature of the floor manifests itself as an exponential relationship between the signal energy and the distance between the sensor and the source. By exploiting this relationship, the authors propose a localization technique that employs the signal energy measurements to estimate the distance between the source and the sensor, rather than solving for the ToA of the waves, or the TDoA among the sensors. In their later work, the authors extend this technique to accomodate the measurement uncertainty. Their Maximum Likelihood Estimation (MLE)-based technique estimates the distance between the source and the sensor by maximizing the likelihood of the observed signal energy measurements [16]. The authors show that their technique outperforms the traditional energy-based techniques in terms of localization accuracy and robustness.

Ambarkutuk et al. [17] propose an information-theoretic approach to mitigate the effects of the dispersion on the localization outcomes. The authors show that measurement uncertainty and dispersion may render some of the sensors as byzantine, i.e., their measurements are not informative, or faulty, with respect to the source location. The authors propose a sensor fusion technique that eliminates the byzantine sensors from the network, and maximizes the joint likelihood of occupant location given the sensor measurements.

MejiaCruz et al. [18] utilize a probabilistic approach to estimate a transfer function and force shape, thereby localizing events based on structural vibrations measurements. Their algorithm, called Probabilistic Force Estimation and Event Localization (PFEEL), involves three main stages: (1) probabilistic transfer function estimation; (2) probabilistic force estimation; and (3) event localization. It uses sensor data to measure vibration waves and applies probabilistic methods to accurately estimate forces and locate events within a structure. In their later work [5], the authors extend the PFEEL algorithm by representing the transfer functions as stochastic processes. This extension allowed the authors to better gauge the uncertainty in the estimated transfer functions and improve the accuracy of the force estimation and event localization.

### 1.2. Challenges in Vibro-Localization

Several challenges exist in the current approaches to vibro-localization:

1. *The sensors are not ideal.* Measurement uncertainty is a crucial factor that contributes to the localization error. The authors of [16] assume a normal distribution of the signal energy measurements to address this. The maximum likelihood solution under the normality assumption yields to a convex optimization landscape. However, due to the complex nature of wave propagation in real-world settings, this assumption rarely holds true. Therefore, the gradient-based solvers may not converge to the global optimum, leading to suboptimal localization results.
2. *The dynamics are complex.* Wave propagation is a complex dynamic phenomenon influenced by various factors, such as dispersion, attenuation, and material inhomogeneities. The authors of [10,19,20] assume a constant wave speed to estimate temporal parameters of the wave, such as the ToA or the TDoA. However, reducing the problem to a single-parameter estimation, i.e., group velocity, may not be sufficient for capturing the complexity of wave propagation in structures. The wave speed can vary depending on several factors, including the material properties of the floor and the frequency of the waves, leading to potential errors in localization accuracy. The authors of [15] assume that the wave speed is solely a function of propagation distance. However, this assumption may oversimplify the complex nature of wave propagation in real-world settings, where wave speed can be influenced by other factors, such as frequency-dependent dispersion and material inhomogeneities.
3. *Each footstep is different.* Ground reaction forces generated by footsteps can vary significantly depending on the individuals’ gait characteristics, such as step length, step width, and walking speed. This variability can introduce errors in the localization results as the technique may not accurately capture the unique characteristics of each individual’s footsteps.
4. *Forcing is unknown*. Vibro-localization techniques often require the estimation of the force shape generated by the occupant’s footsteps [5,18,21]. This force shape is used to estimate the transfer function between forcing and sensor measurements. However, estimating the force shape accurately can be challenging as it depends on various factors, such as the individual’s gait characteristics, the material properties of the floor, and the frequency of the waves. Inaccurate force estimation can lead to errors in the localization results, as the technique may not accurately model the wave propagation through the floor.
5. *The propagation is a rapid phenomenon relative to sampling.* Footsteps generate fast and repetitive signals, leading to a situation where there are insufficient samples to fully capture the wave characteristics at reasonable sampling rates. As a result, transfer function estimates often suffer from low spectral resolution. For instance, [18] employs cross- and auto-spectral density of the measurement vectors to estimate the transfer function of the propagation phenomena. However, the limited frequency resolution and the presence of measurement uncertainty can significantly hinder the accuracy of these estimates and, consequently, the localization results.

### 1.3. Summary of the Contributions

This paper introduces a vibro-localization technique that determines occupants’ locations in a building modeling structural vibration wave propagation, bypassing force estimation. The method simplifies the localization process by employing a model-based approach to represent key propagation phenomena such as dispersion and attenuation, which are parameterized and fitted to measured waveforms. The estimated occupant location is then derived from the joint likelihood function of all sensor measurements.

Our work builds on previous research and makes the following contributions:

- *Multi-Sensor Perception* (addresses challenge 1): We propose a multi-sensor technique that aggregates information from multiple sensors, bypassing the assumption of normal distribution in signal energy measurements. The averaging during the signature estimation does not assume anything about the underlying error distributions, making it more robust to real-world complexities and variabilities.
- *Enhanced Wave Propagation Modeling* (addresses challenges 2, 3, and 4): We propose a model-based approach that accurately captures the dispersive and attenuative properties of structural vibration waves, focusing on key parameters like wave speed and attenuation coefficient. This approach improves localization accuracy by avoiding oversimplifications.
- *A Parametric Approach to Modeling the Physical Properties* (addresses challenge 5): We introduce a piecewise constant velocity profile modeling the dispersion mechanism that can be used to estimate the occupant location, even with low spectral resolution in the transfer function estimates. In the calibration of the vibro-localizer, the wave velocity and attenuation coefficient are estimated by fitting the transfer function to the measured waveforms.

### 1.4. Organization of the Paper

The rest of the paper is organized as follows. In Section 2, we present the proposed vibro-localization and tracking technique. In Section 3, we validate the proposed technique through controlled experiments and field-established datasets. Section 4 presents the results of the validation experiments, providing qualitative and quantitative analyses to evaluate the performance of the proposed technique. Finally, our conclusions are presented in Section 5.

## 2. Methodology

This section presents the proposed vibro-localization technique in greater detail. The localization loss function is derived from the wave propagation model, and the unpropagation operator is used to recover the original waveform.

The proposed technique leverages the structural vibration waves induced by an occupant’s footsteps and captured by a network of sensors distributed throughout the building. The vibro-localization technique estimates the occupant’s location probabilistically by assigning a probability to a location vector, representing the likelihood of the occupant’s presence at that location. The technique is based on our proposed wave propagation model. The concepts of the propagation and unpropagation operators are used to model the wave transmission and recovery processes, respectively. This technique involves estimating the signature waveform induced by the occupant’s footsteps and comparing it with the reconstructed waveforms at the sensors to estimate the occupant’s location.

### 2.1. Forward Problem: Wave Propagation

Consider a homogeneous and isotropic floor surface $S\subset R^{2}$ of a building. When an occupant *O* steps at a location $x_{O}\in S$, they induce a distinct waveform vector $s={\left(s[0],\ldots ,s[K-1]\right)}^{⊤}\in R^{K}$ at that location, where *K* denotes the total number of discrete time samples in the waveform, with each element s[k] corresponding to the recorded signal at time step *k*. This waveform s is referred to as *the signature*, and it propagates through the floor and is detected by stationary sensors placed at various positions $x_{S,i}\in S$, for i∈I={0,…,M−1}.

**Figure 1:**
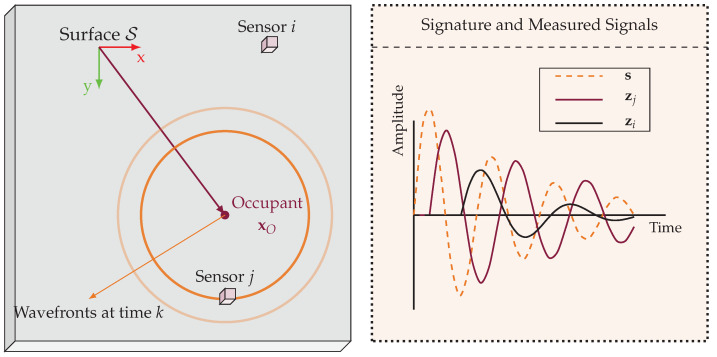
This figure illustrates the wave propagation process. **Left**: An illustration of the geometric layout of the floor, sensors, and the occupant location. **Right**: The wave propagation process from the occupant to the sensors. As can be seen in the figure, both sensors i,j are affected by the wave propagation process. Sensor *i* is further away from the occupant than sensor *j*, which results in a delay and more attenuation relatively to sensor *j*.

The wave propagation process is illustrated in Figure 1. The wave generated by the occupant’s footsteps travels through the floor and is detected by the sensors. For sensor *i*, the observed waveform $z_{i}={\left(z_{i}\left[0\right],\ldots ,z_{i}\left[K-1\right]\right)}^{⊤}\in R^{K}$ is a transformed version of the original waveform due to the effects of propagation. The variations in the observed waveforms $z_{i}$ among different sensors arise from the different propagation paths taken by the wave from the occupant to each sensor.

The transformation in the observed waveform $z_{i}$ is primarily caused by three factors: dispersion, attenuation, and geometric spreading. Dispersion refers to the phenomenon where the phase velocity of the wave varies with its frequency, resulting in a frequency-dependent phase delay τ(ω) in the waveforms. This phase delay can be expressed as follows:

$$\tau \left(\omega \right)=\exp\left(-j\omega \frac{d}{v(\omega )}\right)\;,$$

where $d=∥x_{O}-x_{S,i}∥$ represents the distance between the occupant’s location $x_{O}\left[k\right]$ and the sensor location $x_{S,i}$, and v(ω) is the phase velocity at frequency ω. Attenuation represents the decrease in amplitude of the wave as it travels through the medium. It can be characterized by the following:

$$a=\exp(-\eta_{a}d)\;,$$

where $\eta_{a}$ is the attenuation coefficient. Geometric spreading accounts for the reduction in amplitude due to the wave spreading over a larger area as it propagates. This effect is expressed as follows:

$$g=\left\{\begin{array}{cc} \min\{1,\frac{1}{\sqrt{d}}\} & \mathrm{if}\;d>3\lambda \\ 1 & \mathrm{otherwise}\; \end{array}\right.\;.$$

Together, attenuation *a* and geometric spreading *g* determine how the energy of the wave is distributed and dissipated over the propagation distance *d*. Dispersion, on the other hand, introduces a frequency-dependent phase delay τ(ω) in the waveforms, causing the wave to spread out in time.

To succinctly represent the propagation process, we define the propagation operator *P* and its inverse $P^{-1}$. The operator *P* models the forward wave transmission, while $P^{-1}$ represents the process of recovering the original waveform s from the observed data $z_{i}$. These operators provide a simplified and systematic approach to analyzing waveforms in the vibro-localization technique. The propagation operator *P* is defined mathematically as follows:

$$z_{i}=P\left(s;x,x_{S,i}\right)=a\;g\;F^{-1}\left\{\tau \left(\omega \right)F\left\{s\right\}\right\},$$

where F and $F^{-1}$ represent the Fourier transform and its inverse, respectively. The unpropagation operator $P^{-1}$ is defined as follows:

$$P^{-1}\left(z_{i};x,x_{S,i}\right)=\frac{1}{a\;g}\;F^{-1}\left\{\frac{1}{\tau (\omega )}F\left\{z_{i}\right\}\right\},$$

where the inverse operation recovers the original waveform s from the observed data $z_{i}$.

### 2.2. Inverse Problem: Vibro-Localization

The vibro-localization technique aims to estimate the occupant’s position based on the structural vibrations generated by their footsteps and captured by the sensor network. This method relies on reconstructing the original waveform induced by the occupant’s steps and comparing it with the waveforms observed by the sensors.

**Figure 2:**
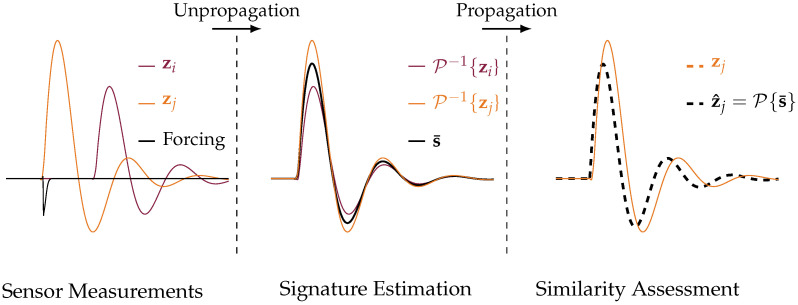
This figure illustrates the process of transforming sensor measurements, $z_{i}$ and $z_{j}$, into estimated signatures and assessing their similarity. The shorthand *P* denotes the propagation operator, which is used to convert sensor data into meaningful signatures. Initially, the unpropagation step transforms the measurements into estimated signatures $P^{-1}\left\{z_{i}\right\}$ and $P^{-1}\left\{z_{j}\right\}$, producing the estimated signature $\bar{s}$. Subsequently, the similarity between the propagated signature $P\{\bar{s}\}$ and the original measurement $z_{j}$ is assessed, allowing for a comparison of the sensor outputs.

As illustrated in Figure 2, the proposed algorithm first computes the average waveform $\bar{s}\left(x\right)$ at each candidate location x using the unpropagation operator.

$$\bar{s}\left(x\right)=\frac{1}{M}\sum_{i\in I}P^{-1}\left\{z_{i},x,x_{S,i}\right\}\;.$$

This average waveform $\bar{s}\left(x\right)$ represents the expected waveform at the candidate location x, averaged over all sensors. Following this, the estimated waveform ${\hat{z}}_{i}\left(x\right)=P\left\{\bar{s}\left(x\right);x;x_{S,i}\right\}$ at each sensor location $x_{S,i}$ is computed by propagating the average waveform $\bar{s}\left(x\right)$ back to the sensor location $x_{S,i}$ for all sensors.

Next, we employ the cosine similarity index between the measurement vector $z_{i}$ and the estimated waveform ${\hat{z}}_{i}\left(x\right)$ to quantify the temporal alignment and scale mismatch between the observed and estimated waveforms. The similarity index, $r_{i}$, which quantifies the agreement between the observed and estimated waveforms at sensor *i*, is defined as follows:

$$r_{i}\left(x\right)=z_{i}\cdot {\hat{z}}_{i}\left(x\right)\;,$$

where · denotes the dot product between two waveforms. Aggregating the similarity indices over all sensors, we obtain the similarity vector $r\left(x\right)={\left(r_{0}\left(x\right),\ldots ,r_{M-1}\left(x\right)\right)}^{⊤}$. Consequently, the similarity vector r quantifies the agreement between the observed and estimated waveforms at each sensor. One might consider the similarity vector r as a feature vector that characterizes the likelihood of the occupant’s presence at a given location x.

An optimization algorithm can be used to solve for the location vector x that maximizes the similarity vector r. We formulate this optimization problem as a probabilistic model, where the similarity index $r_{i}\left(x\right)$ is used to compute the likelihood of the occupant’s presence at each location. The similarity index $r_{i}\left(x\right)$ is evaluated for all locations in the localization space S and then normalized using a softmax function to convert the similarity scores into probabilities, representing the likelihood of the occupant’s presence at each location. The likelihood function $f_{i}\left(x\right)$ at location x for sensor *i* is defined as follows:

$$f_{i}\left(x\right)=\frac{\exp(r_{i}\left(x\right))}{\sum_{x^{\prime }\in S}\exp\left(r_{i}\left(x^{\prime }\right)\right)}\;.$$

Finally, the occupant’s estimated location ${\hat{x}}_{O}$ is determined by maximizing the product of the likelihood functions over all sensors, i.e., the joint likelihood:

$${\hat{x}}_{O}=\underset{x\in S}{\mathrm{argmax}}\prod_{i\in I}f_{i}\left(x\right)\;.$$

This probabilistic approach allows the vibro-localization technique to estimate the occupant’s location by integrating information from multiple sensors, leveraging the unique propagation characteristics of the structural waves in the building.

## 3. Experiments

In this section, we present the objectives and details of the validation studies conducted. The objective is to evaluate the performance of the proposed vibro-localization technique accurately estimating the impact locations, i.e., the occupant locations, by employing the waveform observed by sensors placed at different locations. We consider two datasets representing the wave propagation relevant to the problem of vibro-localization: an aluminum plate and a building-scale experiment. The proposed technique was tested on these datasets to evaluate its performance in estimating the impact locations. Below are the details of the experiments employed in this study.

In the aluminum plate experiment, sensors were strategically placed to ensure uniform coverage across the plate’s surface, facilitating effective calibration and validation of the proposed localization technique. Four piezo-electric sensors were positioned at the corners of the plate to maximize sensitivity to wave propagation patterns while minimizing boundary effects.

In contrast, the building-scale experiment was conducted in Goodwin Hall at Virginia Tech, where sensor distribution was adapted to the structural layout. Sensors were mounted on steel fixtures welded to the I-beams beneath the floor, reflecting real-world constraints. This setup ensured the reliable capture of dispersive wave propagation across a larger and more complex structure.

The distinction between these two experimental configurations highlights two key differences: (1) laboratory-controlled experiments and (2) real-world experiments. This contrast underscores the adaptability and robustness of the proposed technique across varying scales and environments.

### 3.1. Plate Experiment

**Figure 3:**
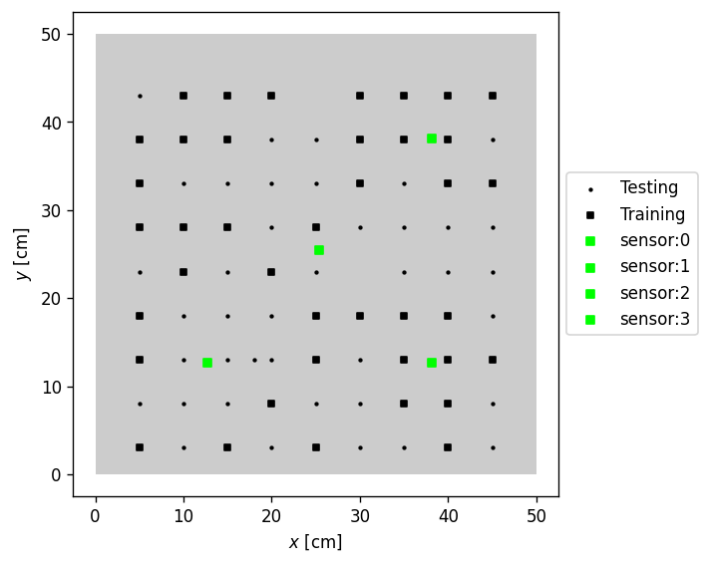
Layout of the aluminum plate experiment, showing impact locations and sensor placements. The 6061 aluminum alloy plate (50 × 50 × 2 cm) was impacted at 81 distinct locations, spaced 5 cm apart. Training points (n=40) are represented by squares (■), while testing points (n=41) are shown as filled circles (●). The positions of four Piezometric PCB 333B50 sensors are marked with green squares (■). This configuration was used to collect vibrational data for calibrating and validating the vibro-localization model.

A 15 kg plate made of 6061 aluminum alloy with dimensions 50 × 50 × 2 cm was tested using the baseline approach [18]. Four Piezometric PCB 333B50 sensors (PCB Piezotronics, Depew, NY, USA) were attached to the plate. The plate was secured at the four corners with 0.40 cm diameter screws, preventing both displacement and rotation. A PCB 086C03, (PCB Piezotronics, Depew, NY, USA), impact hammer struck the plate at eighty-one different locations, spaced 5 cm apart, covering the entire plate area. An instrumented hammer was used to excite the plate, thereby inducing vibrations. Each location was struck once, and force and acceleration data were recorded at a sampling rate of 2048 Hz for 20 s. The experimental data collected from the aluminum plate were divided into two groups. Following the original study, forty out of eighty-one impacts were used to calibrate our model, and the remaining forty-one impacts were used to validate the vibro-localization technique. Figure 3 demonstrates the layout of the sensors and the impact locations on the plate. The training (n=40) and testing points (n=41) were marked with squares (■) and filled circles (●), respectively. The sensor locations were marked with green squares (■).

### 3.2. Building-Scale Experiment with Dispersive Propagation

**Figure 4:**
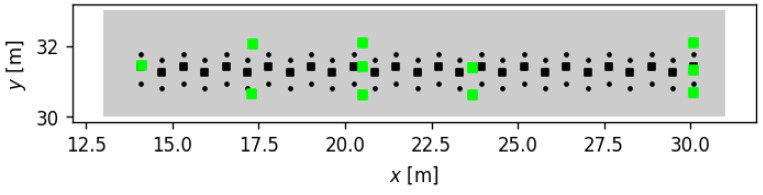
Layout of the experimental setup conducted in Goodwin Hall at Virginia Tech. The figure illustrates the predefined walking path along which participants moved, with step locations marked by black squares (■) for training and black circles (●) for testing. Green squares (■) indicate the sensor locations distributed along the corridor to capture vibrational data. This layout, previously utilized in studies [22,24], serves as the benchmark for building-scale experimental data collection.

The building-scale experimental dataset used in this study was first introduced in [22] and later used in [23,24]. The dataset was collected in the Goodwin Hall at Virginia Tech, a five-story building with a steel frame and concrete floors. This dataset involves participants walking along a predefined path (step length and width were set to 62 and 15 cm) in a corridor, with sensors placed at various locations to capture the vibrations. In these experiments, two participants walked along a predefined 16 m path, separately. Figure 4 shows the step locations—represented by black squares (■) and circles (●), respectively, for training and testing cases—and the sensor locations—represented by green squares (■)—superimposed on the path. We based our reference point on these studies and used the same experimental data as in [24]; thus, it will be referred to as the benchmark in the context of building-scale experiments.

The sensors were mounted on uniform steel mounts welded to the flanges of the structural I-beams beneath the corridor’s concrete floor. Eleven accelerometers that are capable of measuring dynamic out-of-plane acceleration within the frequency range of (2–10,000) Hz and with an average sensitivity of 1000 millivolts per g (where g=9.8 m/s^2^) recorded the structural vibrations. These devices captured data from 162 steps taken by each participant, totaling 324 steps. Data collection was facilitated by EMX-4250 (Ametek VTI Instruments, Irvine, CA, USA) digital signal analyzer cards, connected to the accelerometers via coaxial cables, and equipped with anti-aliasing filters and a high-precision 24-bit ADC. The accelerometer data were sampled at a rate of 1024 Hz. The vibro-localization technique was evaluated using the data collected from the Goodwin Hall experiments. For parameter estimation, the first 27 steps of Occupant A were used to calibrate the model. The model was then tested using the remaining steps from Occupant A and all of Occupant B’s steps. The estimated locations were compared to the ground truth locations to assess the accuracy of the localization.

The training scheme was as follows: the first 27 steps of Occupant A were used to estimate a piecewise linear model representing the phase velocity $v\left(\omega \right)$ of the wave propagation. The model was then used to estimate the impact locations of the remaining steps of Occupant A and all of Occupant B’s steps.

## 4. Results

In this section, we present the results of the proposed technique for both the plate and building datasets. The results are presented in terms of accuracy and precision of the localization estimates. These results are described in detail for each dataset, including the statistical analysis of the localization error and the empirical Probability Density Function (PDF) and Cumulative Distribution Function (CDF) of the localization errors.

### 4.1. Plate Experiments

**Figure 5:**
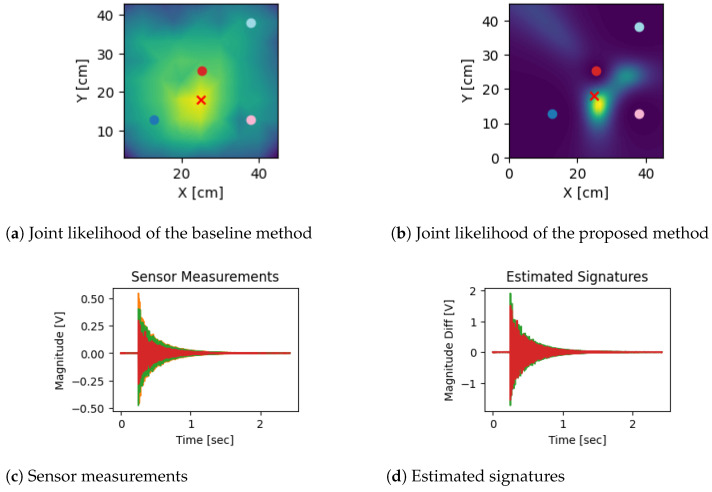
This figure presents a comparison of the joint likelihoods and sensor data for an impact location near the sensor array. The top left subfigure shows the joint likelihood computed using the baseline [18] method, while the top right subfigure displays the joint likelihood obtained from the proposed technique. The bottom left subfigure illustrates the raw sensor measurements, and the bottom right subfigure shows the estimated signatures derived from these measurements. This comparison highlights the performance of both methods in accurately estimating the impact location based on vibrational data.

Figure 5 shows a representative result from the dataset where the impact location is close to the sensor array.

Table 1 provides a detailed statistical descriptives of the localization error such as mean, standard deviation, median, and Mean Absolute Deviation (MAD). The mean and median are measures of accuracy while the standard deviation and MAD are measures of precision, i.e., the spread. The results show that the proposed technique estimated the impact locations with a mean localization error of 18.01 cm and a standard deviation of 1.97 cm. In contrast, the baseline technique reported in [18] exhibited a mean localization error of 19.19 cm with a standard deviation of 7.77 cm. This difference indicates that the proposed technique has shown improvement over the baseline without using the hammer impact information as used in [18].

**Figure 6:**
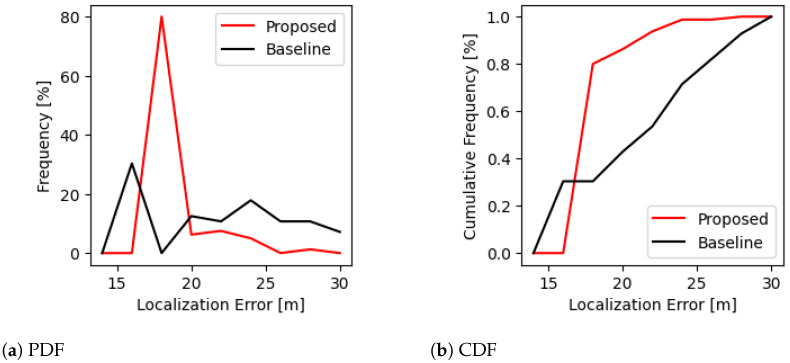
PDF and CDF of localization error for the proposed method and the baseline approach. In the PDF (**left**), the proposed method exhibits a sharp peak around 20 m, indicating a higher frequency of lower localization errors compared to the baseline, which shows a more distributed error profile. The CDF (**right**) further supports this observation, as the proposed method achieves 80% cumulative frequency at a lower error range than the baseline, demonstrating a more consistent and accurate performance. These results suggest that the proposed technique significantly reduces localization error, achieving more reliable estimates than the baseline method.

The empirical PDF and CDF of the localization errors provide further insights into the performance of the proposed technique. Comparing the CDF curves between the proposed and baseline techniques can highlight differences in the overall error distribution. Figure 6 demonstrates the empirical PDF and CDF of the localization errors for the proposed and baseline techniques. As can be seen in the figure, the proposed technique shows a sharper peak in the PDF curve, indicating a higher frequency of small errors relative to the baseline which demonstrated a broader distribution over the range of localization errors. The CDF curve for the proposed technique exhibits a steeper incline at lower error values, suggesting that a significant portion of the errors are small, which is desirable for high-accuracy localization systems. In contrast, the baseline technique shows a more gradual increase in the CDF curve.

### 4.2. Building-Scale Experiments with Dispersive Propagation

**Figure 7:**
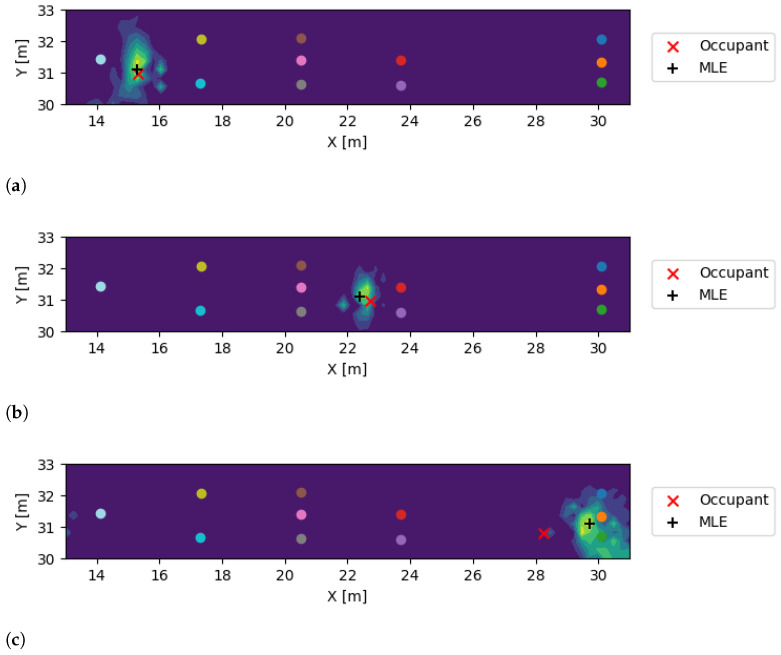
Representative examples of localization results using the proposed method on the building dataset. Each subfigure illustrates the joint likelihood calculated from the measured waveforms, with the true occupant location marked by a red cross (×) and the estimated location by a black plus sign (+). (**a**) shows the occupant at the leftmost end of the corridor, (**b**) at the center, and (**c**) at the rightmost end. These results demonstrate the reliability and effectiveness of the proposed technique in estimating impact locations across different occupant positions.

Figure 7 illustrates three representative results observed in the building dataset. The figure shows the joint likelihood of the proposed technique as well as the estimated impact locations. In an ideal scenario, the joint likelihood function should have a single peak at the true impact location with minimal uncertainty. Figure 7a–c demonstrate a scenario where an occupant is walking along the corridor, and the sensors are placed at different locations to capture the vibrations. Figure 7a demonstrates the scenario where the occupant is located at the leftmost end of the corridor, while Figure 7b shows the scenario where the occupant is located at the center of the corridor. Finally, Figure 7c illustrates the scenario where the occupant is located at the rightmost end of the corridor. The results demonstrate that the proposed technique accurately estimated the impact locations for both occupants.

Localization accuracy was evaluated by comparing the estimated impact locations with the ground truth locations. We employed the mean of the joint likelihood function as the estimated impact location, which was then compared to the ground truth location to calculate the localization error. Table 2 demonstrates the comparative analysis of the proposed and baseline techniques in terms of localization error. The results demonstrate that the proposed technique estimated the impact locations with a Root Mean Squared Error (RMSE) of 0.49 and 0.71 m for Occupants A and B, respectively. On the other hand, the baseline, which was reported in [24], exhibited an RMSE of 0.89 and 0.94 m for Occupants A and B, respectively. This indicates that the proposed technique significantly improves the baseline results in terms of localization accuracy.

Table 3 presents descriptive statistics of localization error for Occupant A and Occupant B using three error estimates considering the outliers in the data: raw, Winsorized at 1 m, and rank-based methods. For the raw data, Occupant A shows a mean localization error of 0.35 m with a standard deviation of 0.21 m, while Occupant B exhibits higher error values, with a mean of 0.62 m and a larger standard deviation of 1.06 m. After Winsorizing at 1 m, Occupant A’s mean error slightly decreases to 0.34 m with a reduction in standard deviation to 0.2 m, and Occupant B’s mean error decreases to 0.48 m with a reduction in standard deviation to 0.29 m. This suggests that extreme values had more influence on Occupant B in the raw data. The rank-based method, which uses median and MAD, provides a more robust central tendency, with Occupant A showing a median error of 0.31 m and an MAD of 0.12 m, while Occupant B has a median of 0.4 m with an MAD of 0.19 m. Additionally, the 95% confidence interval for the mean localization error is (0.31, 0.38) m for Occupant A and (0.44, 0.80) m for Occupant B, indicating that Occupant B consistently shows higher localization error estimates across all metrics, although variability is reduced when outliers are controlled.

**Figure 8:**
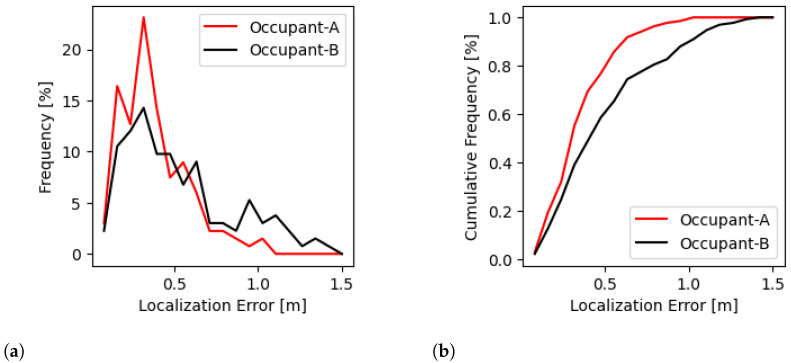
PDF and CDF of the localization error for both occupants in the Goodwin Hall dataset. The results indicate that the error distributions for both Occupant A and Occupant B are similar, demonstrating that the proposed technique is robust to inter-occupant differences. Despite the variations in walking patterns and body dynamics between different individuals, the method maintains relatively consistent performance. (**a**) PDF of the localization error observed in Occupant A and Occupant B data. (**b**) CDF of the localization error observed in Occupant A and Occupant B data.

The empirical PDF and CDF of the localization errors shown in Figure 8 provides additional insights into the performance of the proposed technique. The empirical PDF illustrates the distribution of localization errors, allowing us to observe the concentration of errors around certain values. A sharp peak in the PDF indicates a high frequency of small errors, suggesting that the proposed method consistently achieves close proximity to the true location. Conversely, a broader distribution might indicate greater variability in localization accuracy. The empirical CDF, on the other hand, offers a cumulative perspective by showing the proportion of errors that fall below a certain threshold. A steep incline in the CDF curve at lower error values reflects that a significant portion of the errors are small, which is desirable for high-accuracy localization systems. Comparing the CDF curves between the proposed and baseline techniques aggregates the differences in the overall error distribution, indicating that the proposed method consistently outperforms the baseline across the entire range of localization errors.

## 5. Conclusions

This paper introduces a novel method for characterizing and localizing occupants in a building by analyzing structural vibration waves generated by their activities. The proposed approach utilizes the wave equation, incorporating key properties such as dispersion and attenuation, to propagate and unpropagate structural waves. These properties are estimated from waveforms recorded by sensors placed at various locations in the experimental structure. The experimental validation demonstrates that the proposed method accurately captures the propagation and unpropagation of waves, enabling precise localization of the occupants.

This method is applied to two experimental datasets: a controlled aluminum plate test and a building-scale experiment. The results shows that the proposed technique outperforms existing methods by significantly reducing localization error. For example, in the plate experiment, the proposed method achieves a mean localization error of 18.01 cm with a standard deviation of 1.97 cm, compared to the baseline technique’s mean error of 19.19 cm and standard deviation of 7.77 cm. Similarly, in the building-scale experiment, the proposed method estimates impact locations with an RMSE of 0.49 m for Occupant A and 0.71 m for Occupant B—in contrast to the baseline’s RMSE of 0.89 m and 0.94 m, respectively.

An advantage of the proposed technique is that it does not require prior knowledge of the force exerted by the occupants, and learns the dispersive nature of the floor from the training data. This feature increases the method’s versatility and applicablity to a wider range of scenarios where the force information is not readily available. Also, the proposed method demonstrates its robustness to occupant differences, as similar error distributions are observed for both occupants, underscoring the method’s adaptability to varying walking patterns and footstep characteristics. Vibro-localization is a challenging problem that becomes more difficult as the propagation path becomes longer due to dispersion. With the proposed technique, we are able to accurately estimate the impact locations even in the presence of dispersive propagation, as demonstrated in the building-scale experiment.

In conclusion, this work advances vibro-localization by introducing a model-based approach that accurately represents key propagation phenomena such as dispersion and attenuation. The results demonstrate the technique’s improved accuracy and precision, offering a significant contribution to fields such as structural health monitoring, activity recognition, and security monitoring, where reliable localization of vibration sources is essential.

## Figures and Tables

**Table 1:** Statistical analysis of localization error for the baseline method [18] and the proposed technique. The table compares the accuracy and precision of these techniques. The proposed technique consistently demonstrates lower mean error and variability across all metrics, indicating improved accuracy and robustness compared to the baseline. The reduction in standard deviation and MAD for the proposed method highlights its stability and resistance to outliers in the dataset.

	Raw		Winsorized (at 10 cm)		Rank-Based	
	Mean	B	Mean	Std. Dev.	Median	MAD
Baseline [18]	19.19	7.77	16.34	5.48	19.13	5.43
Proposed	**18.01**	**1.97**	**17.66**	**1.04**	**17.08**	**0.34**

**Table 2:** Comparative analysis of localization error for Occupant A and Occupant B using the proposed and baseline methods. The table presents the RMSE in both the *x*- and *y*-coordinates, as well as the overall magnitude of the RMSE for each position. The proposed method demonstrates a significant reduction in localization error across all metrics for both occupants.

	Occupant A			Occupant B		
	RMSE *x*	RMSE *y*	RMSE ∥·∥	RMSE *x*	RMSE *y*	RMSE ∥·∥
Baseline [24]	0.67	0.58	0.89	0.74	0.58	0.94
Proposed	**0.3**	**0.31**	**0.49**	**0.67**	**0.24**	**0.71**

**Table 3:** Descriptive statistics of localization error for Occupant A and Occupant B using the proposed technique, across different data treatment methods: raw data, Winsorized data (at 1 m), and rank-based data. The results indicate that Occupant A consistently shows lower localization error across all methods compared to Occupant B, with smaller variability. The Winsorized and rank-based methods further reduce the impact of outliers, particularly for Occupant B, where both the mean error and variability are notably reduced. This suggests that the proposed technique is more robust and stable when outliers are controlled, providing more reliable localization estimates.

	Raw		Winsorized (at 1 m)		Rank-Based	
	Mean	Std. Dev.	Mean	Std. Dev.	Median	MAD
Occupant A	0.35	0.21	0.34	0.2	0.31	0.12
Occupant B	0.62	1.06	0.48	0.29	0.4	0.19

## Data Availability

The software realizing the proposed method is developed in Python (version 3.9.12) and is available at http://code.vt.edu/murata/paper-2.

## Abbreviations

The following abbreviations are used in this manuscript:

CDF	Cumulative Distribution Function
MAD	Mean Absolute Deviation
MLE	Maximum Likelihood Estimation
PDF	Probability Density Function
PFEEL	Probabilistic Force Estimation and Event Localization
RMSE	Root Mean Squared Error
TDoA	Time-Difference-of-Arrival
ToA	Time-of-Arrival
